# Modeling contribution of shallow groundwater to evapotranspiration and yield of maize in an arid area

**DOI:** 10.1038/srep43122

**Published:** 2017-02-21

**Authors:** Xiaoyu Gao, Zailin Huo, Zhongyi Qu, Xu Xu, Guanhua Huang, Tammo S. Steenhuis

**Affiliations:** 1 Center for Agricultural Water Research in China, China Agricultural University, Beijing, 100083, P.R. China; 3 Department of Biological and Environmental Engineering, Cornell University, Ithaca, NY, USA; 2 Inner Mongolia Agricultural University, Hohhot, 010018, China

## Abstract

Capillary rise from shallow groundwater can decrease the need for irrigation water. However, simple techniques do not exist to quantify the contribution of capillary flux to crop water use. In this study we develop the Agricultural Water Productivity Model for Shallow Groundwater (AWPM-SG) for calculating capillary fluxes from shallow groundwater using readily available data. The model combines an analytical solution of upward flux from groundwater with the EPIC crop growth model. AWPM-SG was calibrated and validated with 2-year lysimetric experiment with maize. Predicted soil moisture, groundwater depth and leaf area index agreed with the observations. To investigate the response of model, various scenarios were run in which the irrigation amount and groundwater depth were varied. Simulations shows that at groundwater depth of 1 m capillary upward supplied 41% of the evapotranspiration. This reduced to 6% at groundwater depth of 2 m. The yield per unit water consumed (water productivity) was nearly constant for 2.3 kg/m^3^. The yield per unit water applied (irrigation water productivity) increased with decreasing irrigation water because capillary rise made up in part for the lack of irrigation water. Consequently, using AWPM-SG in irrigation scheduling will be beneficial to save more water in areas with shallow groundwater.

In many of semi-arid and arid regions of the world, irrigated agriculture consumes most of the water. For example, in the 1.12 million ha Hetao irrigation district in the Yellow River basin, agricultural irrigation accounts for 90% of the water use^1^,^2^. Especially in districts with shallow groundwater such as the Hetao Irrigation district, capillary rise of groundwater can be used to supplement surface water irrigation and in closed basins can possibly save water for irrigating additional areas.

Several experiments have been carried out to quantify the amount of water that is used by agricultural crops from groundwater. Bargahei and Mosavi reported in an experimental study, groundwater supplemented between 53% and 55% of the water of safflower utilizing saline groundwater and about 82% for safflower utilized fresh groundwater at groundwater depths (between 50 cm to 120 cm)^3^. Ghamarnia found that two thirds of the average annual safflower water requirement (65 cm) could be met with groundwater table depth from 0.6 to 0.8 m^4^. With lysimeter experiments, Kahlown and Ashraf reported that the wheat crop did not need any supplementary irrigation with a water table at 0.5 m depth and 80% sunflower’s water need was supplied by groundwater at 0.5 m depth^5^.

Yield, water productivity (WP) and irrigation water productivity (IWP) vary with both groundwater depth and irrigation amounts. Mejia *et al*. conducted a two-year study to investigate the effect of groundwater table on the yield of corn and found a 5–10% greater yield for corn and 23% for soybeans at 0.5–0.75 m groundwater depth compared with the same treatment with groundwater depth more than 1 m^6^. Huo *et al*. reported that irrigation water productivity (IWP) increased with the shallower groundwater tables^7^.

Although experimental evidence has well established that the shallow groundwater can supplement crop water use, it is seldom used in irrigation scheduling to decrease the amount of water applied because measuring the capillary upward flux is complex and cannot be done routinely. Therefore, models are the only way to obtain the fluxes. However, most of the irrigation management models assume that the groundwater is sufficient deep that the water percolates downward out reach of the plant roots^8^,^9^,^10^. Several methods that can estimate the upward water such as Hydrus and SWAP are cumbersome because they require spatially varying input data such as the soil water retention curve and unsaturated hydraulic conductivity that are not easily available^11^. Prathapar and Qureshi used the SWAP model in Pakistan^12^. Other models such as by Healy and Cook and Ayars *et al*. are based on measured moisture contents or water-table fluctuation and cannot be used in routine application^13^,^14^.

Because of the shortcoming in the above mentioned models for use in irrigation management with shallow groundwater, the objective of this manuscript is, therefore, to develop a quantitative model requiring only readily available data that can be used to analyze the water flux at water table, to calculate the groundwater contribution to crop evapotranspiration; and to investigate the effect of groundwater depth and irrigation water amount on yield, crop evapotranspiration, water productivity and irrigation water productivity of maize. Without affecting the maize yield, the irrigation water can be reduced in a reasonable range. This will aid managers in optimizing water application rates to optimize yields and to determine diversion plans from river systems.

## Model Description

### Agricultural Water Productivity Model for Shallow Groundwater

**Figure 1:**
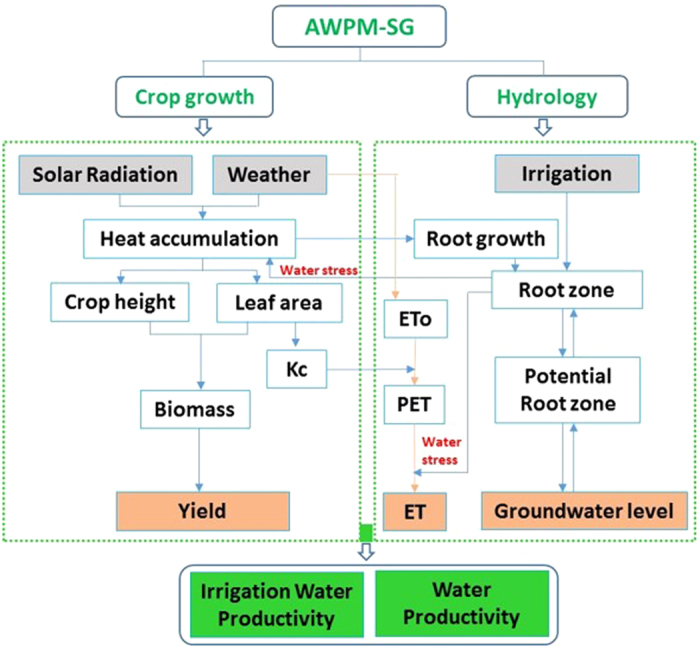
The schematic of the soil water balance calculation and crop growth in the Agricultural Water Productivity Model for Shallow Groundwater (AWPM-SG).

In this study, we develop the Agricultural Water Productivity Model for Shallow Groundwater, AWPM-SG, which is coupling of a crop growth model EPIC (Environmental Policy Integrated Climate) and a soil moisture model of root and vadose zone -WIPE (Watershed Irrigation Potential Estimation) that simulates both upward movement from groundwater and percolation to the groundwater which was originally developed by Saleh *et al*. for surface irrigation in Bangladesh^15^,^16^. The structure of AWPM-SG model is shown in Fig. 1. AWPM-SG model needs few parameters (soil hydraulic parameters and crop growing parameters) listed in Supplementary Tables S1, S2 and S3 in the Section S3 of the Supplementary material and simulates water flux on daily time step. The three parts of the AWPM-SG model consists of a crop module, an actual evapotranspiration module and a soil module (Figure S1 drawn by Xiaoyu Gao in the Section S2 of the Supplementary material). These three modules are coupled first time in the AWPM-SG model. The model is described in detail in the Section S1 of the Supplementary material. An overview of AWPM-SG model is given below.

#### Crop module

The crop module of AWPM-SG is mainly based on the Environmental Policy Integrated Climate (EPIC) model. EPIC originally developed by Williams *et al*.^17^. It has been tested and applied widely around the world^18^,^19^,^20^,^21^,^22^. Wang and Li reported that the EPIC model predicted winter wheat and spring maize yield well on the Loess Plateau in China^23^. Niu *et al*. examined the reliability of the EPIC model in simulating the grain sorghum yields in the USA and found that the accuracy and reliability varied with climate classes and nitrogen treatments^24^. Brown and Rosenberg validated the EPIC model to assess the climate change impact on the potential productivity of corn and wheat in the U.S.^25^. Currently the EPIC model has evolved into a comprehensive model capable of simulating photosynthesis, evapotranspiration and other major plant and soil process^23^. The crop module includes the phonological development, crop growth indexes (LAI, biomass, root growing, crop yield) and water productivity (WP, IWP).

#### Description of soil module

Because EPIC cannot simulate the crop growth above the ground without the soil data, EPIC was coupled with the modified watershed irrigation potential estimation model (WIPE) to simulate the whole water recycle. The WIPE model was designed by Saleh *et al*. to study the impact of irrigation management schemes on groundwater levels in Bangladesh^16^. The model divides the soil profile into four zones namely the actual root zone, potential root zone, transmission zone, and the saturated zone as shown in Figure S1 in the Section S2 of the Supplementary material 16. The soil texture in zone 1 and 2 are same. The zone 1 is calculated using the water balance method. The WIPE model simulates both downward recharge when the soil moisture in zone 2 is greater than field capacity and upward movement when the soil moisture is below field capacity using an analytical solution developed by Gardner^26^. This is a one-dimensional model employing the Thornthwaite-Mather procedure to calculate the recharge below the root zone to the aquifer and is primarily applicable to shallow aquifers^27^. Steenhuis and van der Molen successfully used this model on Long Island, NewYork, USA. Other studies also employed Thornthwaite-Mather procedure^28^,^29^,^30^.

Full detail of the model is shown in the Section S1 (Agricultural Water Productivity Model for Shallow Groundwater) of the Supplementary material.

#### Actual evapotranspiration module

Actual evapotranspiration (ET) is an input to the EPIC model. We used the model of Kendy *et al*. that previously was able to calculate the actual evapotranspiration from soil-water storage in North China Plain with good accuracy^9^. In this model, the ratio of *E*_*p*_ to *T*_*p*_ depends upon the development stage of the leaf canopy, moisture content and root development^9^,^31^,^32^,^33^,^34^. The input data are the daily leaf area index, LAI simulated by the EPIC model and simulated soil moisture of actual root zone (zone 1) by WIPE model and water balance.

## Results

### Evaluation of AWPM-SG model

Data of a lysimetric experimental study carried in 2007 and 2008 at the Shuguang experimental station in the Hetao irrigation district, were used to calibrate and validate the AWPM-SG model. The maize (Kehe-8) commonly grown in Hetao irrigation district was planted in late April and harvested in late September. Supplementary Table S4 in the Section S3 of the Supplementary material shows the irrigation scheduling with four times irrigation every year. Experiments were carried out in two replicates. The groundwater depths were set using the Marriotte bottles at 1.5 m, 2 m, 2.5 m and 3 m before planting. After planting the bottles were removed allowing the groundwater to vary in response to irrigation and evaporation.

The 2007 data were used for calibration and 2008 data for validation (Tables S2 and S3 in the Section S3 of the Supplementary material). The soil data and crop data inputted are shown in Tables S2 and S3 in the Section S3 of the Supplementary material. The goodness of fit test can be found in Table 1 and Table S5 in the Section S3 of the Supplementary material. In case where the observed data were remained nearly the same during the growing season such as the deeper groundwater depth, the Nash Sutcliff efficiency give unrealistic results.

#### Soil moisture: calibration and validation

**Figure 2:**
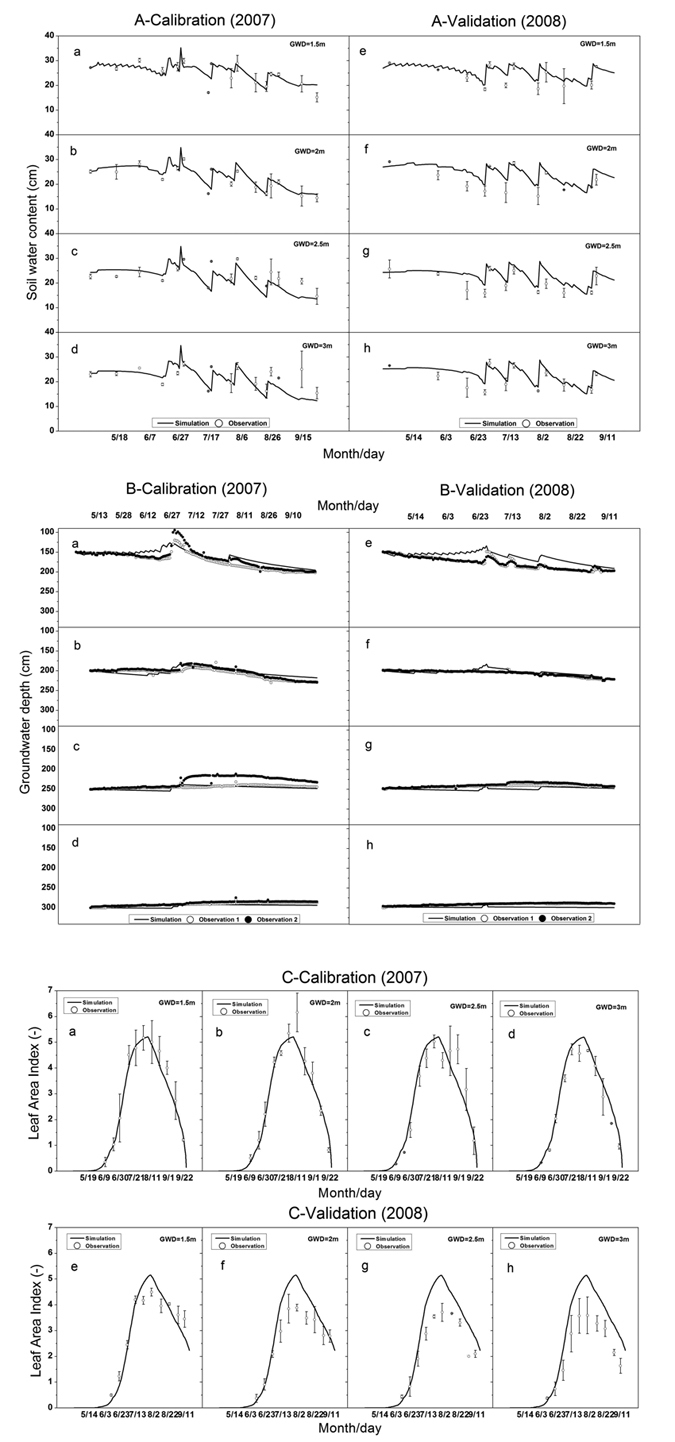
(A,B,C) Simulated versus measured soil water content, groundwater depth and leaf area index under different groundwater depths during calibration (**a–d**) in 2007 and validation (**e–h**) in 2008. A is that simulated versus measured soil water content, B is that simulated versus measured groundwater depth and C is that simulated versus measured leaf area index.

The soil water content of top 90 cm (zones 1 and 2) was used to calibrate and validate the model. Simulated mean soil water content in 2007 in the 90 cm soil zone for groundwater depth at 150 cm and 200 cm were generally satisfactory simulated with the Nash and Sutcliffe model efficiency (NSE) of 0.57, 0.68 and R^2^ of 0.57, 0.69. (Figure 2A(a,b)). For deeper water table depth at 250 and 300 cm, the simulated water content fitted visually well (Figure 2A(c,d)) and the goodness of fit was reasonable for most statistics presented in Table 1. In 2008 the soil moisture was well predicted with the calibrated input data (Fig. 2A(e–h), Table 1) including the NSE that varied from 0.39 to 0.74 and R^2^ varying from 0.77 to 0.88. (Table 1). For the calibration and validation, the averaged RMSE value was 2.97 cm varying from 2.09 cm to 4.16 cm.

#### Groundwater table: calibration and validation

Figure 2B show that the AWPM-SG simulations of the groundwater table followed the same trend with observations during the calibration period as indicated by the goodness of fit parameters with the R^2^ around 0.6 and averaged RMSE value of 9.6 cm (Table 1 and S5). For the calibration period the simulated data in 2008 (Fig. 2B) had a similar accuracy with the MRE varying from −8.28% to 3.28%. (Table 1).

#### Crop leaf area index (LAI): calibration and validation

Figure 2C(a–d) shows that the simulated LAI (calculated with Equation S3 and S6 in the Section S1 of the Supplementary material) followed the same trend as the observations during the calibration period. The NSE for LAI varied from 0.88 to 0.97 and R^2^ varied from 0.88 to 0.98 indicating a good fit (Table 1). The averaged RMSE value for calibration and validation was 0.6 cm^2^/cm^2^. The measured LAI in 2008 was less than in 2007, which can have many causes that were not included in the model such as the low temperature and snow in seeding stage as well as different observers in 2007 and 2008. LAI was therefore not as well predicted by AWPM-SG for the validation period as for the calibration period with the NSE ranging from 0.18 to 0.88 (Fig. 2C(e–h)).

#### Parameter sensitivity and uncertainty analysis

To investigate the effect of calibrated parameters on the crop evapotranspiration, groundwater depth, LAI and soil water content of 90 cm soil, the parameters sensitivity and uncertainty were analyzed together (Figure S2). When the parameters vary, the four indexes are linearly related to the change in the parameters basically except for the T_b_, PHU and C. For the crop evapotranspiration, the LAI_mx_ affects the ET most obviously, and then the be and T_b_ largely affect the ET (Figure S2A and B). The other parameters affect the ET slightly with the ΔET/ET less than 5% as the parameters increasing by 25%. Evenly most of the parameters have little impact on the ET. The initial LAImx is from the recommended value of EPIC. Through the calibration and sensitivity of parameters the final LAI is determined. The detailed information is shown in Figure S2 in the Section S2 of Supplementary material.

For the groundwater depth, the mf, dp, T_b_ and PHU are the main impact factors. The others almost have no influence on the groundwater depth (Figure S2C and D). mf is measured in the field experiment using undisturbed samples. The initial dp is obtained by the recommended value of local government and T_b_, PHU is obtained by the recommended value of EPIC. The detailed information is shown in Figure S2 in the Section S2 of Supplementary material.

Similarly to the ET, the main impact factors on LAI are LAI_mx_, T_b_, PHU, T0 and ad. The other parameters have little effect on the LAI. The initial T_b_, PHU, T0 and ad are recommended by EPIC. The detailed information is shown in Figure S2 in the Section S2 of Supplementary material.

For the soil water content of 90 cm, the parameters such as mf, ms, mwp, LAI_mx_, PHU, T_b_ affect the soil water content more obviously than the other parameters. Mf, ms, mwp are measured in the experiment. The initial LAI_mx_, PHU, T_b_ are recommended by EPIC. The detailed information is shown in Figure S2 in the Section S2 of Supplementary material.

Combing the observed data, the uncertainty analysis of the parameters on groundwater depth, LAI and soil water content are analyzed (Table S6 in the Section S3 of Supplementary material). Through the uncertainty analysis of parameters, the *d-factors* calculated by Equation (7) ranges from 0 to 1.44 with averaged value of 0.14. Larger d-factor represents larger uncertainty. From the data, we can find that for the groundwater depth, LAI and soil water content, the most uncertain parameters are same with the most sensitive parameter. The most uncertain parameters are mf, LAI_mx_, mf for groundwater depth, LAI and soil water content, respectively. The trend of uncertainty of parameters are basically similar to the sensitivity. The detailed information is shown in Table S6 in the Section S3 of Supplementary material.

#### Water fluxes and capillary rise under different groundwater depths

In this model the water flux at the boundary of zone 2 (RD <  = RD_mx_) or zone 1 (RD = RD_mx_) is the water flux at water table. In this model we assume the water flux at 90 cm (maximum root) as the groundwater contribution to crop water use. In the analysis, groundwater depth is the initial groundwater condition for different groundwater depth. The soil water flux obtained by adding the percolation (calculated with Equation S23 in the Section S1 of Supplementary material) and capillary rise (Equation S24 in the Section S1 of Supplementary material) during the growing period from April 20^th^ to September 24^th^ for 2007 for groundwater tables from 150 to 300 cm are shown in Figure S3 in the Section S2 of the Supplementary material. There were eighteen recharge events for 150 cm groundwater depth and only four when the groundwater was at 2 m or deeper. The root zone is wetter when the groundwater is shallow (Fig. 2A) and the soil is brought up to field capacity faster than when the groundwater is deeper. Assuming that all irrigation or rain water in excess of field capacity percolates downward, this means that there is more recharge for the shallow groundwater tables. For example, with the irrigation of 9.75 cm irrigation on June 26^th^ in 2007, the groundwater table rose 53 cm for the 150 cm depth and 1 cm for water table at 300 cm (Figure S3 in the Section S2 of the Supplementary material). Without the rainfall and irrigation, water from groundwater will move upward to the root zone. The net groundwater contribution (i.e., upward minus recharge) to soil root zone for whole crop growth stage was 106 mm (24%) when groundwater depth was at 1.5 m, while recharges was 51 mm with groundwater depth of 3 m. Our modeling results are similar to Huo *et al*., where the groundwater capillary flow comprised 29% of the water use of wheat at 1.5 m groundwater depth from planting to harvest and decreased with an increase in table depth^7^.

### Scenarios for evaluation of water use and yield

After calibration and validation of AWPM-SG, the model was used to find out the response of groundwater depth and deficit irrigation on water use, maize yield, WP and IWP. To do so the AWPM-SG was run for nine scenarios with 7 GWD levels (100, 150, 200, 250, 300, 350 and 400 cm). D0 (irrigation with 360 mm, 53% of PET), D1 (irrigation with 288 mm, 42% of PET), D2 (irrigation with 270 mm, 40% of PET), D3 (irrigation with 240 mm, 35% of PET), D4 (irrigation with 180 mm, 26% of PET), D5 (irrigation with 120 mm, 18% of PET), D6 (irrigation with 90 mm, 13% of PET), D7 (irrigation with 72 mm, 11% of PET) and D8 (no irrigation) were simulated (Table 2). The rainfall of 128 mm for all scenarios and the irrigation for the D0 treatment was similar to the amount in 2007 used for calibration of the model. The initial soil water, groundwater depth, irrigation time and fertilizer application are set as the lysimeter experiments of groundwater at 1.5 m in 2007. The initial meteorology of the model is done by repeated meteorology in the experiments in 2007. In the analysis for the effect of groundwater on water flux, evapotranspiration and yield, groundwater depth is the initial groundwater condition for different scenarios.

#### Evapotranspiration of maize

**Figure 3:**
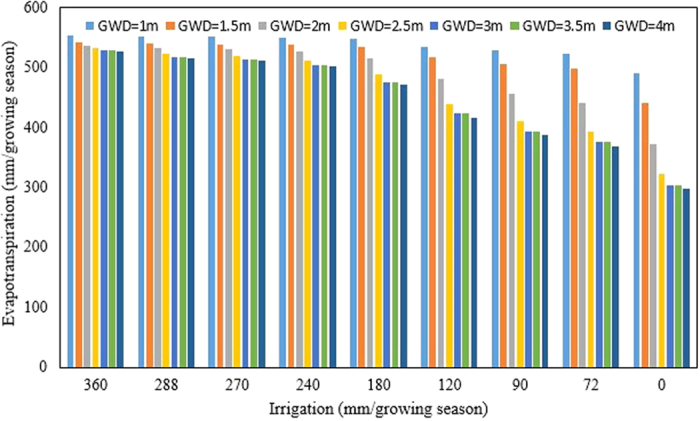
Actual evapotranspiration of maize during the growing period with groundwater depth ranging from 1 m to 4 m and irrigation amounts ranging from 0 to 360 mm.

The scenario analysis found that evapotranspiration (calculated by Equation S32 in the Section S1 of the Supplementary material) was the greatest for the shallowest groundwater depth at 1 m amounted to around 570 mm per growing season and was nearly independent of the amount of irrigation water applied (Fig. 3). The initial soil moisture content was 0.3 cm^3^/cm^3^ and rainfall during the growing season was 128 mm for the all scenarios. Evapotranspiration decreased gradually when the groundwater depth increased from 1 to 4 m depth. For groundwater depth of 1 m, the evapotranspiration was around 550 mm independent of the amount of irrigation applied. When the groundwater is at 4 m, less water evaporated when irrigation amounts decreased and amounted to 300 mm per growing season when the groundwater was at 4 m depth and the maize crop was not irrigated (Fig. 3).

#### The groundwater contribution to crop evapotranspiration (WF/ET)

**Figure 4:**
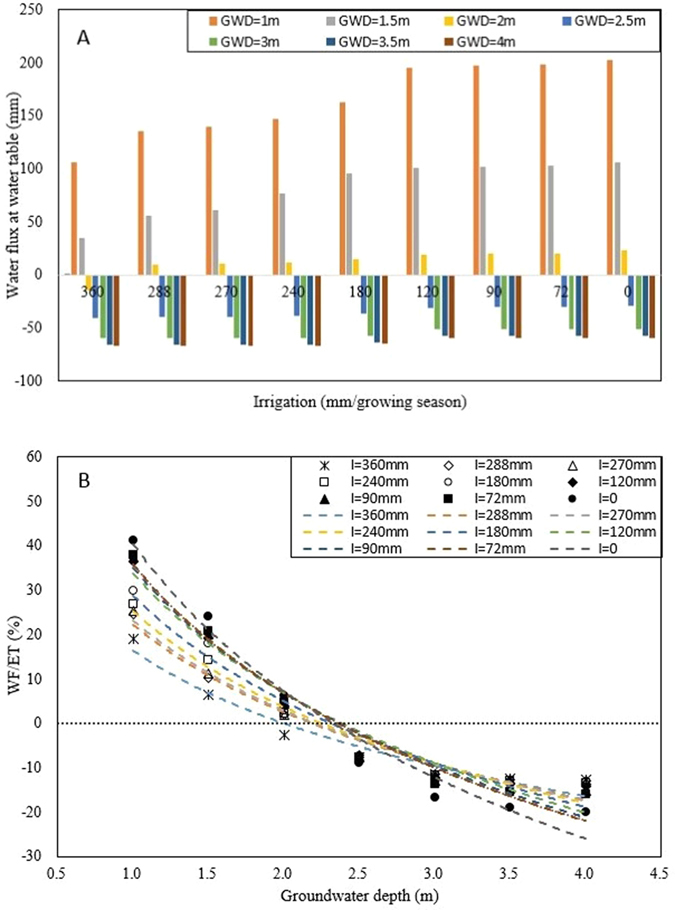
Water fluxes at water table and groundwater contribution to water use (WF/ET) under various groundwater depths and irrigation amounts. Note: A is that water fluxes at water table; B is that groundwater contribution to water use (WF/ET); GWD is groundwater depth; Irrigation is the total amount of irrigation applied during the growing season. Individual irrigation amounts are listed in Table 2. WF is the net water flux at water table, ET is the actual evapotranspiration.

The predicted water flux at water table for various irrigation treatments and groundwater depths are shown in Fig. 4A. Within the crop growth period, root zone will become deeper. At the same time, groundwater levels will change. So flux between groundwater and root zone is dynamic and depend on distance between water table and the rooting depth. For the same groundwater depth, the upward water flux increases as the irrigation amount becomes smaller, especially for the shallow groundwater depth (Fig. 4A). For example, at 1 m groundwater depth, the upward water flux was 202 mm without irrigation and nearly half of the amount evapotranspirated. The result show that under shallow groundwater, groundwater contribution can be considered in the irrigation system. When the groundwater table was at 2 m depth, there was a net recharge of −14 mm in the growing season when 360 mm of irrigation water was applied (Fig. 4A) and without irrigation there was a 23 mm of upward flux. Finally, with groundwater at 4 m, 67 mm of the 360 mm water applied is lost from the root zone. Thus when the groundwater depth is more than 2.5 m, upward movement is negligible as can be seen in Fig. 4A.

#### Maize yield under different groundwater depth and different irrigation scenarios

The yield maize (calculated with Equation S10 in the Section S1 of the Supplementary material) is almost unaffected when the groundwater is shallow depths with more irrigation water applied (Table 3). For example, for the D0 treatment (irrigation at 53% of PET with 360 mm), the yield of maize is 12.44 tons/ha for groundwater at 1 m depth and 12.42 tons/ha for groundwater at 4 m depth. However, when groundwater is more than 2 m deep irrigation amounts are directly related to the crop yield. Without irrigation, the yield was 12.42 tons/ha with groundwater at 1 m water table depth but reduced by approximately 1/2 to 7.0 tons/ha for groundwater at 4 m depth (Table 3).

#### Water productivity and irrigation water productivity

Water productivity (WP) (calculated with Equation S12 in the Section S1 of the Supplementary material) is the ratio of crop yield to crop evapotranspiration. Irrigation water productivity (IWP) (calculated with Equation S13 in the Section S1 of the Supplementary material) is the ratio of crop yield to irrigation amount (Table 3). The WP is nearly constant for all irrigation amounts and all groundwater depth and thus the yield is directly related to the amount of evapotranspiration. It should however be noted that this is only true for deficit irrigation where less irrigation water is applied than the potential evapotranspiration during the summer. The WP is increasing slightly with less irrigation at the same groundwater depth. For the same irrigation application with more than 180 mm, WP is increasing with deeper groundwater.

**Figure 5:**
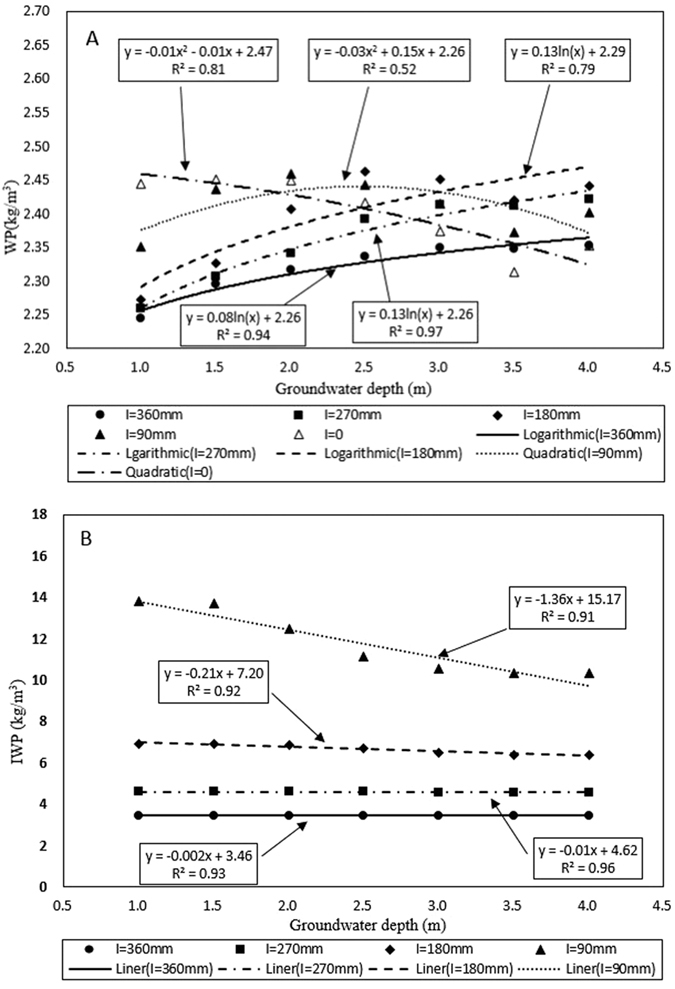
Relationship between water productivity, irrigation productivity and groundwater depth. Note: A-Relationship between water productivity and groundwater depth. B-Relationship between irrigation water productivity and groundwater depth.

The IWP increased with decreasing water application (Table 3, Fig. 5). The averaged IWP value for seven groundwater levels for the D0 (irrigation at 53% of PET), D2 (irrigation at 40% of PET), D4 (irrigation at 26% of PET) and D6 (irrigation at 13% of PET) irrigation treatments were 3.4, 4.5, 6.5 and 11.8 kg/m^3^. This clearly shows the positive effect of water table depth on maize yield.

## Discussion

In order to simulate the irrigation water management models in arid areas with shallow groundwater, the Agricultural Water Productivity Model for Shallow Groundwater (AWPM-SG) (Fig. 1) was developed for the simulation of the water fluxes, deep percolation and capillary rise as a function of groundwater depth. The model needs few parameters (soil hydraulic parameters and crop growing parameters) and simulates water flux on daily time step. The model was calibrated and validated using the lysimeter experimental data in 2007 and 2008. The simulation of soil moisture, groundwater depth and LAI resulted in reasonable agreement, which indicated that the model is suitable to simulate water fluxes and water productivity in the shallow groundwater district.

The water flux at water table, crop evapotranspiration and groundwater contribution were investigated with running AWPM-SG in scenarios where the irrigation amount and water table depth was varied. Evapotranspiration was nearly independent of the amount of water applied when the groundwater was shallow and for groundwater below 1.5 m decreased with smaller amounts of irrigation water (Fig. 3 and Table 3). Thus the capillary rise can make up for the lack of irrigation water. This is agreement of the findings of Soppe and Ayars^35^, the irrigation applied was 46% less when the water table was maintained at 1.5 m depth than when the water table was too deep to be reached by the crops, while our results are that ET only decreased by 3% with irrigation applied 50% less at 0.5 m water table depth when ET decreased by 9% with irrigation applied 50% less at 3 m water table depth (Fig. 3).

We found that the quotient of groundwater flux at water table (WF) and the evapotranspiration (ET) increases with both decrease in irrigation amounts and groundwater depth (Fig. 4B). This is similar to the findings of Luo and Sophocleous that ratios of seasonal groundwater evaporation to seasonal potential evapotranspiration are plotted against the depth to water table^36^. Our results (Fig. 4) are in general agreement with experimental results of Torres and Hanks and Luo and Sophocleous that up to 2.5 m depth of groundwater table upwards movement occurs and for deeper depth the groundwater contribution is almost negligible^36^,^37^. For example, in the Luo and Sophocleous’s experiments in the sub-humid warm temperate continental monsoon Yucheng, China, only 3% of the evapotranspiration was supplied by groundwater at 3 m depth^36^.

Under deficit irrigation, maize yield is directly linked to crop evapotranspiration (i.e. WP ≈ 2.2) and almost independent of the amount of irrigation applied for shallow groundwater tables (Table 3). Consequently, using AWPM-SG in irrigation scheduling together with groundwater monitoring will increase the yield per unit of water applied in irrigation district with high groundwater. The IWP of maize slightly decreased when groundwater depth increased with linear relationship and significantly increased with less irrigation amount (Fig. 5). Thus, these simulations agree with earlier findings of Huo *et al*. and Sun *et al*.^7^,^38^, the amount of water productivity per unit of water applied for shallow groundwater can be improved with moderate deficit irrigation. In addition, the deeper the groundwater level, the WP will be more different between different irrigation treatments. For closed basin where the amount of irrigation water is limiting, applying less water will increase the overall yield per unit area in the basin.

Future improvement to the model will include modules that can simulate other crops. In addition, we will add a module that simulates the movement of salt so that irrigation manager can more effectively leach the salt from the profile.

## Materials and Methods

### Experiments

For model calibration and validation, field experiments by Kong were used^39^. Investigating the effect of water table depth (ranging from 1.5 m to 3 m) on water use efficiency of maize using lysimeters at the Shuguang Experimental farm in the Hetao irrigation district. The experimental station located at open field and have a representative climate, soil, groundwater conditions. Lysimetric experiments provide accurate water balances and have been widely used to validate models^9^,^36^,^40^.

The Hetao irrigation district, is located in the western part of Inner Mongolia Autonomous Region. The study site has a typically arid and semi-arid continental climate. The average annual rainfall is142 mm and falls mainly from June to August. The average annual pan evaporation is 2300 mm. The average number of sunlight hours per month is 266 h. The mean annual temperature is 7 °C, with monthly averages of −10.1 in January to 23.8 °C in July (Figure S4 in the Section S2 of the Supplementary material). Soils begin to freeze in the second half of November to a maximum depth of about 1.0–1.3 m, and is completely thawed in the middle of May. Groundwater depth varies between 1.2 to 3.8 m^41^.

Maize, wheat and sunflower accounts for 31%, 7% and 30% respectively. These proportions have varied very little in recent years^42^. The experiment with eight non-weighing lysemeters grown with maize were carried by Fanrui Kong in 2007 and 2008 in the middle of the experimental field with groundwater depth initially at 1.5 m, 2 m, 2.5 m and 3 m replicated two times^39^. The iron cylindrical lysimeters had a diameter of 1 m and a height of 3.35 m. The maize (Kehe-8) commonly grown in Hetao was planted in late April and harvested in late September. Cultivation practices were similar to the practices by farmers and recommended by extension agents. Four times during the growing season 360 mm of water was applied (Table S4 in the Section S3 of the Supplementary material). The irrigation amount referred to the local farmers’ field. Nitrogen fertilizers were applied at a rate of 207 kg/ha at the first irrigation, 103 kg/ha at the third irrigation in 2007 and 276 kg/ha at the first irrigation in 2008. Marriotte bottles were used to set the initial groundwater depths at 1.5 m, 2 m, 2.5 m and 3 m before sowing, and then removed to allow the groundwater to change in response to irrigation and evaporation.

During the growing period, the meteorological data consisting of air temperature, sunshine hours, relative humidity and wind speed for 2007 and 2008 were taken from Linhe weather station 8 km away from Shuguang and were used to calculate the FAO Penman-Monteith reference evapotranspiration (ET_0_). Daily rainfall was measured using a rain gauge at the experimental site. The rainfall was 132 mm in 2007 and 123 mm in 2008, with more than 70% of precipitation occurring in July and August. The main weather data was shown in Figure S4 in the Section S2 of the Supplementary material.

The particle size distribution was obtained using the laser particle analyzer. The main soil physical properties of the soil are shown in Supplementary Table S1 in the Section S3 of the Supplementary material. In the root zone, the soil is loam, which is the representative soil texture in this region^42^. The dry bulk density was obtained by oven drying undisturbed soil samples of 100 cm^3^ at 105 °C for 48 h. Saturated hydraulic conductivity was measured in eight 100 cm^3^ undisturbed soil samples using a constant-head permeameter^43^. Soil water retention curves were determined for each horizon in 100 cm^3^ undisturbed soil samples using a pressure membrane apparatus (SEC-1000). The fitted soil hydraulic parameters of the Muchlem-van Genuchten model are presented in Supplementary Table S3 in the Section S3 of the Supplementary material 44,45.

### Data

The soil moisture content was measured every 3–17 days using the time domain reflectometry (Tube-TDR) including shortly before and after the irrigation and at seeding and harvest. The soil moisture was measured at 20 cm intervals from the surface to a depth of 300 cm. At the same time, it was calibrated with drying method through earth-fetching at regular time.

The groundwater depth was monitored daily during the crop growth period through monitoring the piezo metric head in the access chamber.

The crop leaf area index (LAI) was measured every 6–12 days using a leaf area meter (LI-3000, LI-COR).

Dry maize yield was determined after harvesting.

### Model calibration and validation

The simulation period for maize was from late April to late September in 2007 and 2008 using the observed initial soil water content and groundwater depth subject to the imposed irrigation schedule and fertilizer applications for two years. The 2007 data was used for calibration and 2008 to validate the model. Soil moisture of top 90 cm (zone 1 and 2), groundwater depth, and LAI were simulated. The soil hydraulic parameters (*md, ms, C, α* and *ks*) and the crop parameters were calibrated (Tables S2 and S3 in the Section S3 in the Supplementary material). During the calibration, we set the parameters od model according to the measured data and recommended values, and we analyzed the sensitivity and uncertainty analysis of parameters and found the sensitive parameters for soil water, groundwater depth and crop LAI, respectively such as LAI_mx_, mf and so on. Then we adjust the parameters as their sensitivity to make the simulation result of model more close to measured data. At last the calibrated parameters were used to validate the model using the data of 2008. The default values of the EPIC model for maize were used as initial value for simulating crop growth^46^. The default value of maximum rooting depth was 90 cm measured in the experiment by Kong^39^. Initial soil water content and groundwater depth were specified according to measurements. A sensitivity and uncertainty analysis of parameters was performed for soil water content, groundwater depth and crop LAI.

The upper boundary condition was determined by the actual evaporation and transpiration rates, and the irrigation and precipitation fluxed. A no-flux boundary condition was specified at the column bottom.

The mean relative error (MRE), the root mean square error (RMSE), the Nash and Sutcliffe model efficiency (NSE), the coefficient of determination (R^2^) and the coefficient of regression (b) were used to quantify the model-fitting performance for both calibration and validation processes. These indicators were defined as follows^40^,^47^:

MRE is the mean relative error. The MRE close to 0 indicates good model predictions.


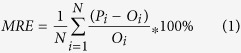


RMSE is the root mean square error. The RMSE value close to 0 indicates good model predictions.


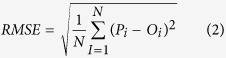


NSE is the Nash and Sutcliffe model efficiency. NSE = 1.0 represents a perfect fit, NSE close to 0 represents the predicted values near to the averaged measurement, and negative NSE values indicate that the mean observed value is a better predictor than the simulated value


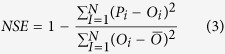


R^2^ is the coefficient of determination. R^2^ value close to 1 indicates good model predictions.


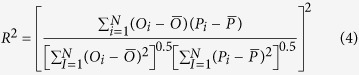


b is the coefficient of regression. b value close to 1 indicates good model predictions


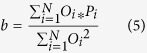


Where N is the total number of observations, *P*_*i*_ and *O*_*i*_ are respectively the ith predicted and observed values (i = 1, 2, …, N), and 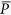 and 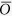 are the predicted and observed mean values, respectively^48^.

### Sensitivity analysis of parameters

Through increasing or decreasing of the percentage of the parameters, the variation of ET, groundwater depth, LAI and soil water of 90 cm are analyzed. Then the sensitivity of each parameter on ET, groundwater depth, LAI and soil water of 90 cm can be obtained.

### Uncertainty analysis of parameters

The *d-factor* was used to analyze the uncertainty of parameters^49^,^50^. The *d-factor* is indicative of average distance between the upper and lower confidence interval (in this study the 95% prediction interval). The *d-factor* was calculated as follows:


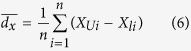



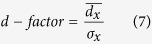


Where 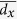 is the average distance between the lower *X*_*L*_ and the upper limits *X*_*U*_ of the confidence interval and *σ*_*x*_ is the standard deviation of observed data. n is the number of data. Larger *d-factors* will lead to larger uncertainty.

### Data availability

The data for this paper are available in the observation section in the Supplementary materials. The data consists of the observed soil moisture, groundwater depth and leaf area index for two years; the maximum, minimum and average temperature during the growing season for two years; the daily rainfall, wind speed in 2007 and 2008 and the calculation of reference evapotranspiration for two years.

## Additional Information

**Publisher's note:** Springer Nature remains neutral with regard to jurisdictional claims in published maps and institutional affiliations.

## Supplementary Material

Supplementary Material

## Figures and Tables

**Table 1:** Mean relative error, Root mean square error, Regression coefficient, Nash and Sutcliffe model efficiency and Coefficient of determination of the model.

	Calibration (2007)				Validation (2008)		
	GWD (cm)	Soil water content	Groundwater depth	LAI	Soil water content	Groundwater depth	LAI
Mean relative error, MRE (%)	150	1.77	−2.13	−2.72	6.2	−8.28	−7.68
	200	2.67	1.46	−0.81	10.8	−0.52	12.63
	250	−8.38	4.11	8.75	12.5	3.21	22.78
	300	−7.05	1.92	15.04	5	2.58	33.89
Root mean square error, RMSE (cm/cm^2^ cm^−2^)	150	2.93	12.39	0.3	2.09	17.5	0.45
	200	2.7	8.97	0.46	3.03	5.29	0.7
	250	4.16	10.9	0.59	3.05	8.57	0.89
	300	3.7	6	0.36	2.11	8.05	1.01
Regression coefficient, b	150	0.98	0.97	0.95	1.04	0.91	1.05
	200	1	1.01	0.93	1.07	0.99	1.21
	250	0.91	1.04	0.98	1.1	1.03	1.3
	300	0.93	1.02	1.08	1.02	1.03	1.4
Nash and Sutcliffe model efficiency, NSE	150	0.57	0.67	0.97	0.72	−0.53	0.88
	200	0.68	0.58	0.94	0.57	0.44	0.62
	250	−0.01	−0.66	0.88	0.39	−4.42	0.37
	300	0	−0.97	0.95	0.74	−7.64	0.18
Coefficient of determination, R^2^	150	0.57	0.72	0.98	0.88	0.61	0.93
	200	0.69	0.64	0.95	0.77	0.56	0.96
	250	0.44	0.4	0.88	0.79	0.14	0.96
	300	0.51	0.66	0.98	0.79	0.01	0.97

Note: GWD is groundwater depth.

**Table 2:** Irrigation amount and time at groundwater depth being 1, 1.5, 2, 2.5, 3, 3.5 and 4 m (mm).

	6/26	7/17	8/1	8/22	Total
Experiment (D0, 53% of PET)	97.5	90	97.5	75	360
4/5 experiment (D1, 42% of PET)	78	72	78	60	288
3/4 experiment (D2, 40% of PET)	73.1	67.5	73.1	56.3	270
2/3 experiment (D3, 35% of PET)	65	60	65	50	240
1/2 experiment (D4, 26% of PET)	48.75	45	48.75	37.5	180
1/3 experiment (D5, 18% of PET)	32.5	30	32.5	25	120
1/4 experiment (D6, 13% of PET)	24.4	22.5	24.4	18.8	90
1/5 experiment (D7, 11% of PET)	17.5	18	17.5	15	72
0 (D8)	0	0	0	0	0

Note: PET is the potential evapotranspiration calculated by LAI in the model. D0 refers to the experimental irrigation with amount of irrigation applied being 53% of PET; D1-D8 represent the deficit irrigation. D1–4/5 of experimental irrigation with amount of irrigation applied being 42% of PET; D2–3/4 of experimental irrigation with amount of irrigation applied being 40% of PET; D3–2/3 of experimental irrigation with amount of irrigation applied being 35% of PET; D4–1/2 of experimental irrigation with amount of irrigation applied being 26% of PET; D5–1/3 of experimental irrigation with amount of irrigation applied being 18% of PET; D6–1/4 of experimental irrigation with amount of irrigation applied being 13% of PET; D7–1/5 of experimental irrigation with amount of irrigation applied being 11% of PET; D8-no irrigation.

**Table 3:** Maize yield, evapotranspiration (ET), water productivity (WP) and irrigation water productivity (IWP) for different groundwater depth and irrigation treatments.

Irrigation treatment	Groundwater depth(m)	Y	ET	I	WP(Y/ET)	IWP(Y/I)
		(t/ha)	(mm)	(mm)	(kg/m^3^)	(kg/m^3^)
S	1.0	12.44	554	360	2.24	3.46
	1.5	12.43	542	360	2.29	3.45
	2.0	12.43	536	360	2.32	3.45
	2.5	12.42	532	360	2.34	3.45
	3.0	12.42	529	360	2.35	3.45
	3.5	12.42	529	360	2.35	3.45
	4.0	12.42	528	360	2.35	3.45
D2	1.0	12.44	551	270	2.26	4.61
	1.5	12.43	539	270	2.31	4.60
	2.0	12.42	531	270	2.34	4.60
	2.5	12.41	519	270	2.39	4.59
	3.0	12.38	513	270	2.41	4.59
	3.5	12.37	513	270	2.41	4.58
	4.0	12.37	511	270	2.42	4.58
D4	1.0	12.44	547	180	2.27	6.91
	1.5	12.43	534	180	2.33	6.90
	2.0	12.38	514	180	2.41	6.88
	2.5	12.05	489	180	2.46	6.70
	3.0	11.67	476	180	2.45	6.48
	3.5	11.53	476	180	2.42	6.40
	4.0	11.49	471	180	2.44	6.38
D6	1.0	12.43	529	90	2.35	13.81
	1.5	12.34	507	90	2.44	13.71
	2.0	11.22	456	90	2.46	12.47
	2.5	10.03	411	90	2.44	11.15
	3.0	9.50	394	90	2.41	10.56
	3.5	9.33	394	90	2.37	10.37
	4.0	9.29	387	90	2.40	10.32
D8	1.0	11.99	490	0	2.44	
	1.5	10.79	440	0	2.45	
	2.0	9.11	372	0	2.45	
	2.5	7.80	323	0	2.42	
	3.0	7.22	304	0	2.37	
	3.5	7.04	304	0	2.31	
	4.0	7.00	297	0	2.35	

Note: PET is the potential evapotranspiration calculated by LAI in the model. S refers to the experimental irrigation with amount of irrigation applied being 53% of PET; D1–D8 represent the deficit irrigation. D1–4/5 of experimental irrigation with amount of irrigation applied being 42% of PET; D2–3/4 of experimental irrigation with amount of irrigation applied being 40% of PET; D3–2/3 of experimental irrigation with amount of irrigation applied being 35% of PET; D4–1/2 of experimental irrigation with amount of irrigation applied being 26% of PET; D5–1/3 of experimental irrigation with amount of irrigation applied being 18% of PET; D6–1/4 of experimental irrigation with amount of irrigation applied being 13% of PET; D7–1/5 of experimental irrigation with amount of irrigation applied being 11% of PET; D8-no irrigation.
