# Supplementary material for: Modeling contribution of shallow groundwater to evapotranspiration and yield of maize in an arid area
Source: Sci Rep. 2017 Feb 21;7:43122. doi: 10.1038/srep43122 (PMC5318869; doi:10.1038/srep43122)
Supplement: Supplementary Material [file srep43122-s1.pdf]

Supplementary material

Title of manuscript: Modeling contribution of shallow groundwater to evapotranspiration and yield of maize in an arid area

Authors: Xiaoyu Gao[1, 3], Zailin Huo[1*], Zhongyi Qu[2], Xu Xu[1], Guanhua Huang[1], Tammo S. Steenhuis[3*]

[1] Center for Agricultural Water Research in China, China Agricultural University, Beijing, 100083, PR China

[2] Inner Mongolia Agricultural University, Hohhot, 010018, China

[3] Department of Biological and Environmental Engineering, Cornell University, Ithaca, NY, USA

Corresponding authors

Zailin Huo

Center for Agricultural Water Research in China, China Agricultural University

No.17 Qinghua East Road, Haidian, Beijing, 100083, PR China

Phone: +86-10-62736762; fax: +86-10-62736762; email: huozl@cau.edu.cn

Tammo S. Steenhuis

Department of Biological and Environmental Engineering,

Cornell University, Ithaca, NY, USA

Email: tammo@cornell.edu

Section S1 Agricultural Water Productivity Model (AWPM-SG)

I. Description of crop module-EPIC

II. Description of soil module-WIPE

III. Description of actual evapotranspiration

Section S2 Figures

Figure S1 Schematic of the soil profile used in the AWPM-SG

Figure S2 Parameters sensitivity analysis for ET, groundwater depth, LAI and soil water content of top 90 cm.

Figure S3 Water flux at water table (simulated values), Precipitation, Irrigation and groundwater depth (GWD) during the growth period (observed values) in 2007

Figure S4 Average minimum and maximum temperature and rainfall during the crop growing period during the two years that the lysimeter experiment was carried in Shuguang experimental site where it was arid and semi-arid climate

Section S3 Tables

Table S1 Soil physical properties of experimental area

Table S2 Default and calibrated values of maize's physiological parameters for the crop growth part of AWPM-SG

Table S3 Values of the hydrological parameters

Table S4 Irrigation scheduling carried out for the experiments of maize in Shuguang village in 2007 and 2008

Table S5 Mean relative error, Root mean square error, Regression coefficient, Nash and Sutcliffe model efficiency and Coefficient of determination of the model calibration and validation

Table S6 Uncertainty analysis of parameters for AWPM-SG

Section S1: Agricultural Water Productivity Model for Shallow Groundwater (AWPM-SG)

AWPM-SG model overview

AWPM-SG incorporates a new crop growth model on WIPE's original code. It combines the WIPE model (Saleh et al., 1989) with EPIC crop growth model (Williams et al., 2006) to simulate the hydrological processes completely including the soil water flow, water flux at water table and crop growing. The WIPE-EPIC model requires various inputs for soil (field capacity, permanent wilting point, residual moisture, saturated water content, saturated hydraulic conductivity), groundwater (drainable porosity), daily weather (minimum and maximum temperature, solar radiation, rainfall), crop (crop-specific base temperature, value of the maximum of crop leaf area index, parameter that governs LAI decline rate for crop, the value of HUI when LAI starts declining, harvest index), management (irrigation, growing time) and initial conditions (volumetric soil water content, groundwater depth). The AWPM-SG model needs less demanding data input soil parameters to simulate the groundwater capillary when compared with the SWAP-EPIC model by Xu et al. (2015). The schematic of the AWPM-SG is shown in Figure 2.

Description of crop module

The EPIC plant growth model was developed to estimate soil productivity as affected by erosion throughout the U.S. The processes simulated include leaf-area index; conversion of biomass; above ground mass; economic yield; root growth and water use.

Phonological development

In EPIC, phonological development of the crop is based on daily heat unit accumulation. It is computed using the equation

$$HU_K = \left(\frac{T_{mx,K}+T_{mn,K}}{2}\right) - T_b \qquad HU_K > 0 \quad \text{(S1)}$$

Where *HU*, *Tmx*, *Tmn* are the values of heat units, maximum temperature and minimum temperature in °C for any day K, and T_b is the crop-specific base temperature in °C. A heat unit index (HUI) ranging from 0 at planting to 1 at physiological maturity is calculated as follows.

$$HUI_i = \frac{\sum_{K=1}^{i} HU_K}{PHU} \quad \text{(S2)}$$

Where *HUI* is the heat unit index for day i and *PHU* is the potential heat units required for maturity. The value of *PHU* may be provided by the user or calculated by the model from normal planting and harvest dates.

Potential growth

LAI is simulated as function of heat units, crop stress and crop development stages. From emergence to the start of leaf decline, LAI is estimated with the equation

$$\mathrm{LAI}_i = LAI_{i-1} + \Delta LAI \quad \text{(S3)}$$

$$\Delta\mathrm{LAI} = (\Delta\mathrm{HUF})(LAI_{mx})(1 - \exp[5(LAI_{i-1} - LAI_{mx})])(REG_i)^{0.5} \quad \text{(S4)}$$

$$\mathrm{HUF}_i = \frac{HUI_i}{HUI_i + \exp(ab_1 - ab_2(HUI_i))} \quad \text{(S5)}$$

Where *HUF* is the heat unit factor, *REG* is the value of the minimum crop stress factor. LAI_{mx} is the value of the maximum of crop leaf area index, ab_1, ab_2 are the crop parameters.

From the start of leaf decline to the end of the growing season, LAI is estimated with the equation

$$\mathrm{LAI}_i = LAI_0 (\frac{1-HUI_i}{1-HUI_0})^{ad} \quad \text{(S6)}$$

Where ad is a parameter that governs LAI decline rate for crop and HUI_0 is the value of HUI when LAI starts declining.

Root growth

Root length is simulated as function of the heat units and the maximum of root length. The root length will be maximum before the mature stage.

$$RD_i = RD_{i-1} + \Delta RD \quad \text{(S7)}$$

$$\Delta RD_i = 2.5 * RD_{mx} * (\Delta HUF) \qquad RD_i \leq RD_{mx} \quad \text{(S8)}$$

$$RD_i = RD_{mx} \qquad RD_i \geq RD_{mx} \quad \text{(S9)}$$

Where ΔRD_i is the variation of root depth in the ith day (cm); RD_i is the root depth in the ith day (cm); RD_{mx} is the maximum depth reached by roots (cm).

Crop yield and water use efficiency

The crop yield considering the water stress can be expressed as the equation.

$$\mathrm{YLD} = \mathrm{HI}_{adj} * B_a \quad \text{(S10)}$$

$$HI_{adj} = \frac{HI}{1+WSYF(0.9-WS)\max\{0,\sin[\frac{\pi}{2}(\frac{HUI-0.3}{0.3})]\}} \quad \text{(S11)}$$

Where *WSYF* is a parameter expressing the sensitivity of harvest index to drought, *WS* is the water stress factor. *HI* is the harvest index. Ba is the crop biomass above ground and calculated by LAI and BE (a conversion factor of crop transferring the energy to biomass).

Water productivity and irrigation water productivity was computed using the following equation:

$$\mathrm{WP} = \mathrm{Y/ET} \quad \text{(S12)}$$

$$\mathrm{IWP} = \mathrm{Y}/\mathrm{I} \tag{S13}$$

Where WP represent WUE, water use efficiency (kg/m^3), and IWP represent irrigation water use efficiency (kg/m^3). Y denotes the final grain yield (kg/ha), ET is the total ET (mm), I is the irrigation amount from planting to harvest (mm).

The input parameters of crop module (Figure 1 and Supplementary Table S2 in the Section 3 of the supplementary materials) include daily maximum, minimum and mean value of temperature, T_{max}, T_{min} and T_{mean} (^{o}C); crop-specific base temperature, T_b (^{o}C); the potential heat units required for maturity, *PHU*; the maximum of crop leaf area index, LAI_{max}; the maximum of crop root, RD_{mx}; the crop parameters, ab_1, ab_2; the parameter that governs LAI decline rate for crop, *ad*; the value of HUI when LAI starts declining, HUI_0; the parameter expressing the sensitivity of harvest index to drought, *WYSF*; the harvest index, *HI* (Supplementary Table S2 in the Section 3 of the supplementary materials). The output is daily leaf area index, LAI; crop height, *h*; root growth, *R*; crop yield, *Y and water productivity, WP, IWP*.

Description of soil module

The underground model in this study is a modification of the watershed irrigation potential estimation (WIPE) model designed by Saleh et al. (1989) to study the impact of irrigation management schemes on groundwater levels. This is a one-dimensional model employing the Thornthwaite-Mather procedure to calculate the recharge (Steenhuis and van Molen, 1985) of the aquifer and is primarily applicable to shallow aquifers. Precipitation, irrigation, soil properties such as the moisture content and the hydraulic conductivity and the initial groundwater level are required to run this model.

The model begins by dividing the soil profile into four zones namely the current root zone, future root zone, transmission zone, and the saturated zone over an impermeable bed as shown in Figure S1 in the supplementary materials. The zone 1 is the zone occupied by roots; the zone 2 is the zone that is not currently occupied by the roots but will be so after their complete development; the zone 3 is the unsaturated transition zone below the root zone with lower boundary at water table and the thickness of this layer varies in time according to extraction/evaporation and recharge; the zone 4 is the saturated zone and is regarded as the water table. The zone 3 is always at the constant moisture content and is equal to the saturated moisture content minus the drainable porosity.

Soil water flow

When $\mathrm{RD}_i \leq RD_{mx}$, *water balance is calculated in the zone 1*

$$\mathrm{CR(i)} = \frac{\mathrm{mg} * (RD_{mx} - \mathrm{RD}_i) * (\mathrm{RD}_i - \mathrm{RD}_{i-1})}{RD_{mx} - \mathrm{RD}_{i-1}} \quad \text{(S14)}$$

When $\mathrm{mr} * \mathrm{RD}_i \geq \mathrm{mf} * \mathrm{RD}_i$ *where mf is field capacity*

$$\mathrm{ET} = ET_p \quad \text{(S15)}$$

$$\mathrm{CAP} = 0 \quad \text{(S16)}$$

$$P_w = \mathrm{P} + \mathrm{I} + \mathrm{CAP} + \mathrm{CR} + \mathrm{mr} * \mathrm{RD} * 10 - \mathrm{mf} * \mathrm{RD} * 10 - \mathrm{ET} \quad \text{(S17)}$$

Otherwise:

$$P_w = 0 \quad \text{(S18)}$$

$$D_r = \mathrm{D0} * \exp[\mathrm{b} * \frac{(mr - mwp) + (mg - mwp)}{2}] \quad \text{(S19)}$$

$$\mathrm{CAP} = 100 * D_r * \frac{(mg - mwp) - (mr - mwp)}{0.5 * RD_{mx} * 10} \quad \text{(S20)}$$

$$Wr_{i+1} = Wr_i + P + I + CAP + CR - ET - P_w \quad \text{(S21)}$$

$$mr_{i+1} = Wr_{i+1} / (RD_{i+1} * 10) \quad \text{(S22)}$$

Where Wr is water content in the zone 1(mm); mr, mg are the soil moisture in the zone 1, 2 (cm^3/cm^3); P is precipitation (mm), I is irrigation (mm), ET is actual evapotranspiration (mm); CR is the water depth supplied to the root zone from deeper zone due to the root growth (mm); P_w is the water depths that leave the current root zone (mm); CAP is the capillary rise from zone 2 (mm) (Ritchie, 1996) , D_r is the averaged diffusivity of zone 1 (cm^2/day); D0 is the diffusivity at wilt pointing of zone 1 (cm^2/day); b is the empirical parameter of soil. In the study, the soil texture of zone 1 and zone 2 are same.

Water balance calculation of zone 2:

When *mg*⩾*mf* (field capacity) where *mf* is the redistribution moisture content of root zone and the flux J is given by (Saleh et al., 1989)

$$\mathrm{J} = (RD_{mx} - \mathrm{RD}) * \{\mathrm{mg} + \frac{ms-md}{C} * \ln\left[\frac{C*k_2s*\exp(-C)}{d*(ms-md)} + \exp\left(-C * \frac{mg-md}{ms-md}\right)\right] - md\} \quad \text{(S23)}$$

Which is always directed downwards (mm). Here *ms* is the saturated moisture content of root zone (cm^3/cm^3), *md* is the air dry moisture content of root zone (cm^3/cm^3), k_2s is the saturated hydraulic conductivity of root zone (mm/day), and *C* is a constant to 13. In this condition, there will be no upward evaporation flux from the aquifer so flux=-J. When *mg*<*mf* there will not be any downward flux so that J=0. However, the upward evaporative flux from the aquifer will be non-zero and is a function of depth to water table from soil surface as given by Gardner (1958)

$$\mathrm{u} = \mathrm{ks} * (\frac{e^{-\alpha\varphi}-1}{1-e^{\alpha h}}) \quad \text{(S24)}$$

Where *ks* is the saturated hydraulic conductivity of transmission zone (mm/day), *h* is the depth to the water table (mm), α is the diffusivity coefficient which is the inverse

of air entry φ_h, and φ is the matric potential calculated as

$$\varphi = \varphi_h \left\{\exp\left[5 * \left(1.13 - \frac{m-md}{ms-md}\right)\right] - 0.93\right\} \quad \text{(S25)}$$

The air entry value is calculated using (Saxton et al 1986)

$$\varphi_h = 100 * [-0.108 + 0.341 * ms] \quad \text{(S26)}$$

When water table is closer to soil surface the flux will be maximum and as water table goes down the flux will decrease. The limiting depth at which the flux becomes zero is approximately 4.5 meter below ground level.

Irrigation using groundwater is simulated by extracting water from the aquifer and adding it to root zone. The water table depth is updated as

$$h_{t+\Delta t} = h_t - \left(\frac{1}{dp}\right)(J - u - ex_t) * \Delta t \quad \text{(S27)}$$

Where ex_t is the extraction rate and *dp* is the drainable porosity.

$$Wg_{i+1} = Wg_i + P_w - CAP - CR - J + u \quad \text{(S28)}$$

$$mg_{i+1} = Wg_{i+1}/(RD_{mx} * 10 - RD_{i+1} * 10) \quad \text{(S29)}$$

Where Wg is water content in the zone 2 (mm); mg is the soil moisture in the zone 2 (cm^3/cm^3).

When $\mathrm{RD}_i > RD_{mx}$, there will be no zone 2, the calculation of zone 1 is similar to zone 2 when $\mathrm{RD}_i \leq RD_{mx}$.

The input parameters of soil module (Figure 1 and Supplementary Table S3 in the Section 3 of the supplementary materials) include Saturated moisture, ms; Air-dry moisture, *md*; Saturated hydraulic conductivity, Ks, constant C, b; the thickness of zone 1 and 2 (maximum root depth), the diffusivity at wilt pointing , D0; initial soil moisture and groundwater depth. The output is daily soil moisture and groundwater depth.

Description of actual evapotranspiration module, ET_a

The actual evapotranspiration is calculated and subtracted from soil-water storage. ET_a is a fraction of potential evapotransiration, ET_p, which consists of potential evaporation from soil, E_p, and potential transpiration from plants, T_p. The ratio of E_p to T_p depends upon the development stage of the leaf canopy, expressed as τ, the dimensionless fraction of incident beam radiation that penetrates the canopy (Campbell and Norman, 1998, p.249)

$$\tau = \exp[(-\mathrm{kb}) * \mathrm{LAI}] \quad \text{(S30)}$$

kb is the dimensionless canopy extinction coefficient, with a value of about 0.82 (Stockle, 1985) and *LAI* is leaf-area index, daily values of which can be obtained by simulation of EPIC.

Accordingly, ET_p is allocated to

$$E_p = (\tau)(ET_p) and T_p = (1-\tau)(ET_p) \quad \text{(S31)}$$

Actual evapotranspiraton, ET (mm), can be limited by the availability of water in the soil. Thus total actual evaporation and transpiration from soil are modeled as (Rawls and Brakensiek, 1985; Maidment 1993), Kendy et al. (2003) calculate the crop evapotranspiration using this method in the north of China.

$$E_a = E_p \left[1 - \left(\frac{mr}{mwp}\right)^{-be}\right] and T_a = T_p[1 - (\frac{mr}{mwp})^{-bt}] \quad \text{(S32)}$$

$$\mathrm{ET} = E_a + T_a \quad \text{(S33)}$$

Where *mr* is the moisture content of root zone and *bt*=3 for transpiration and *be*=0.8 for evaporation.

Section S2

Figure captions

Figure S1 Schematic of the soil profile used in the AWPM-SG

Figure S2 Parameters sensitivity analysis for ET, groundwater depth, LAI and soil water content of top 90 cm.

Figure S3 Water flux at water table (simulated values), Precipitation, Irrigation and groundwater depth (GWD) during the growth period (observed values) in 2007

Figure S4 Average minimum and maximum temperature and rainfall during the crop growing period during the two years that the lysimeter experiment was carried in Shuguang experimental site where it was arid and semi-arid climate

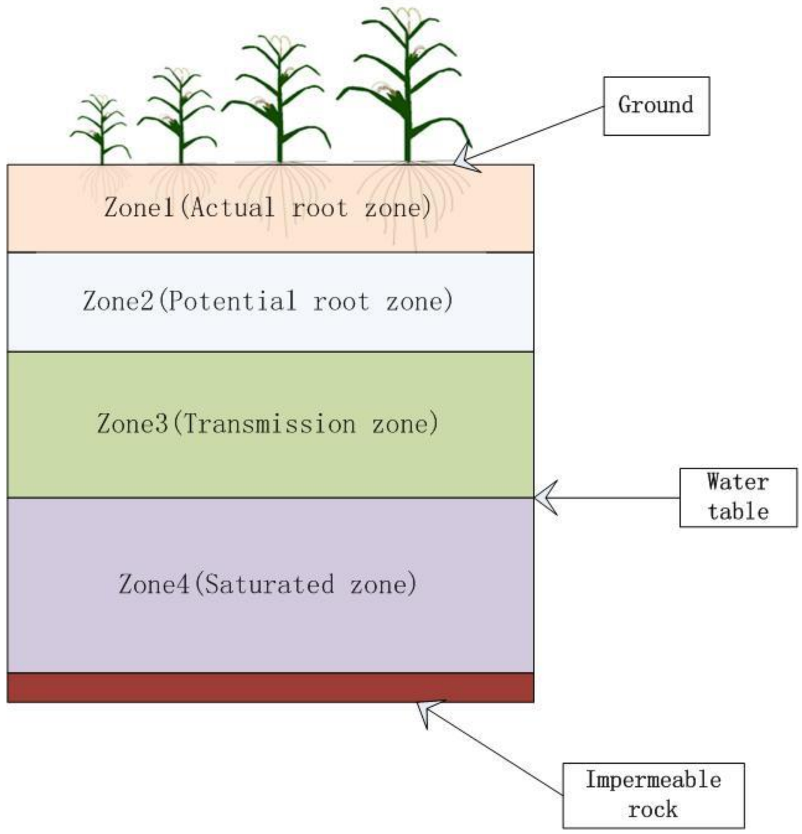

Figure S1 Schematic of the soil profile used in the AWPM-SG

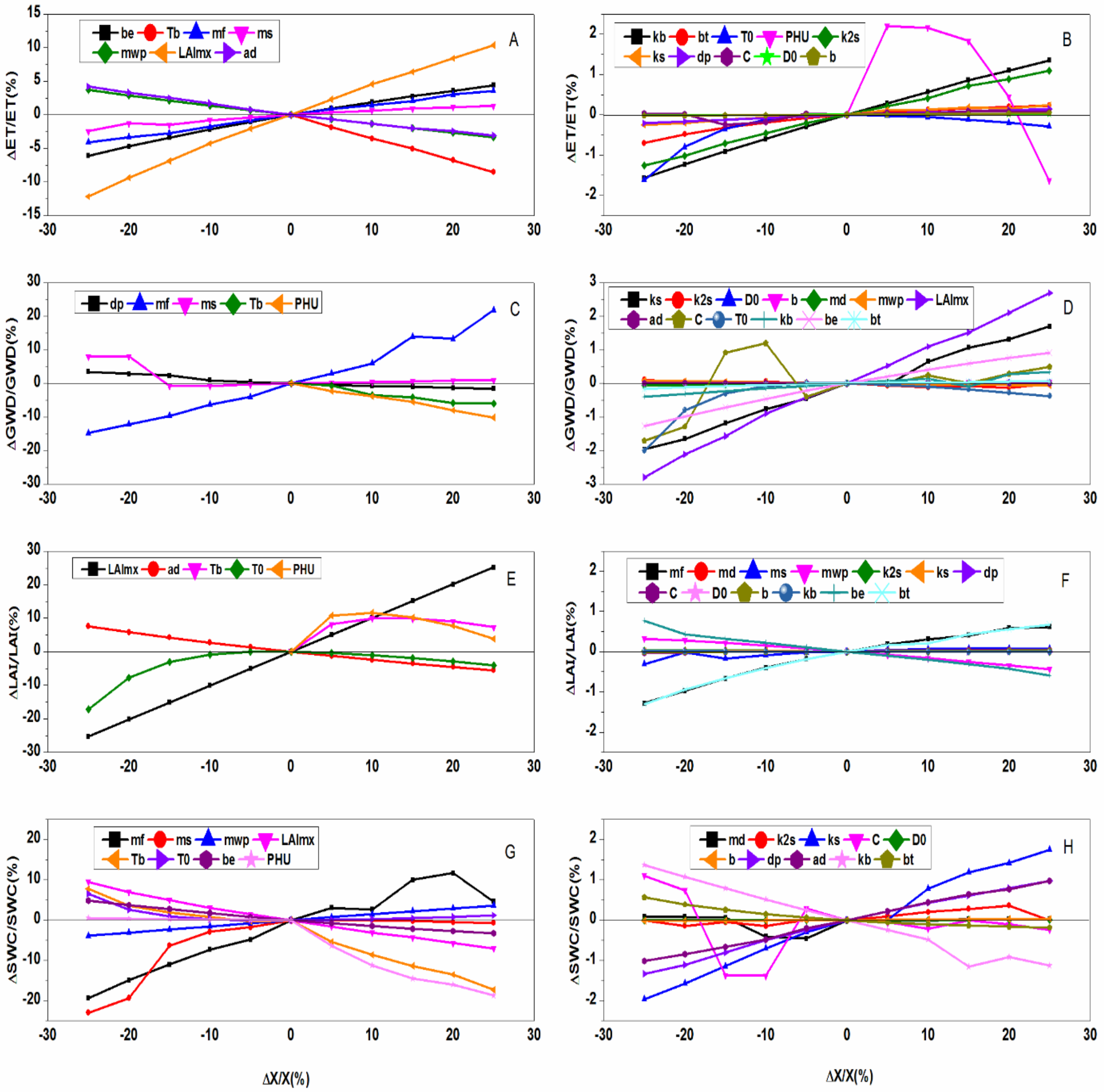

Figure S2 Parameters sensitivity analysis for ET, groundwater depth, LAI and soil water content of 90 cm.

Note: A is the more sensitive parameters for ET (LAImx, ad, be, Tb, mf, ms, mwp), B is the less sensitive parameters for ET (kb, bt, T0, PHU, k2s, ks, dp, C, D0, b); C is the more sensitive parameters for groundwater depth (dp, mf, ms, Tb, PHU), D is the less sensitive parameters for groundwater depth (ks, k2s, D0, b, md, mwp, LAImx, ad, C, T0, kb, be, bt); E is the more sensitive parameters for LAI (LAImx, ad, Tb, T0, PHU), F is the less sensitive parameters for LAI (mf, md, ms, mwp, k2s, ks, dp, C, D0, b, kb, be, bt); G is the more sensitive parameters for soil water content (mf, ms, mwp, LAImx, Tb, T0, be, PHU), H is the less sensitive parameters for soil water content (md, k2s, ks, C, D0, b, dp, ad, kb, bt).

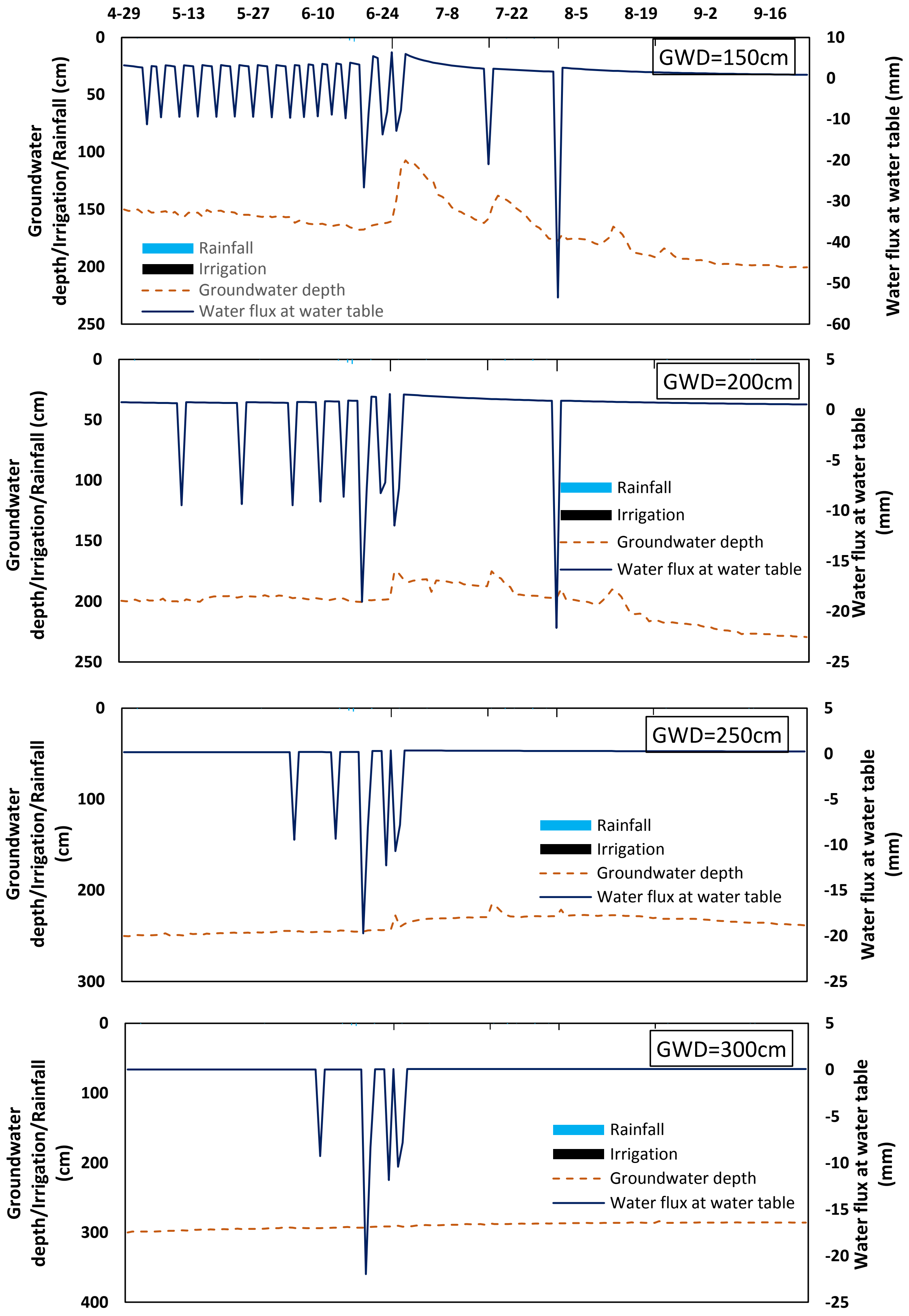

Figure S3 Water flux at water table (simulated values), Precipitation, Irrigation and groundwater depth (GWD) during the growth period (observed values) in 2007

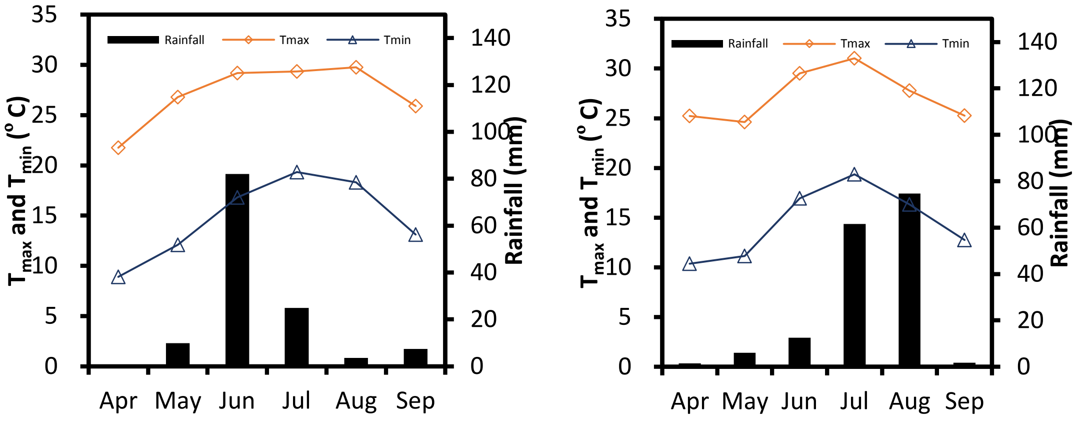

(2007) (2008)

Figure S4 Average minimum and maximum temperature and rainfall during the crop growing period during the two years that the lysimeter experiment was carried in Shuguang experimental site where it was arid and semi-arid climate

Section S3

Table captions

Table S1 Soil physical properties of experimental area

Table S2 Default and calibrated values of maize's physiological parameters for the crop growth part of AWPM-SG

Table S3 Values of the hydrological parameters

Table S4 Irrigation scheduling carried out for the experiments of maize in Shuguang village in 2007 and 2008

Table S5 Mean relative error, Root mean square error, Regression coefficient, Nash and Sutcliffe model efficiency and Coefficient of determination of the model calibration and validation

Table S6 Uncertainty analysis of parameters for AWPM-SG

Table S1 Soil physical properties of experimental area

Soil depths (cm)	Soil particle size distribution			Soil texture	Bulk density (g/cm^3)	Field capacity, *mf* (cm^3/cm^3)	Wilting point (cm^3/cm^3)
	Clay (<0.002mm)	Silt (0.002-0.05mm)	Sand (0.05-2.0mm)				
0-90	17.8	29.4	52.8	Loam	1.27	0.32	0.04
90-150	8.1	39.1	52.8	Sandy loam	1.32	0.30	0.06

Table S2 Default and calibrated values of maize's physiological parameters for the crop growth part of AWPM-SG

Parameters	Default values	Calibrated values
dimensionless canopy extinction coefficient, kb	0.5	0.2
be	0.3	0.8
bt	4	3
Minimun temperature for plant growth, T_b (^oC)	8	8
Optimal temperature for plant growth, T_0 (^oC)	25	25
Leaf area index decline rate, *ad*	1.0	0.5
Maximum crop height, h_{mx} (cm)	200	225
Maximum leaf area index, LAI_{mx}	6.0	5.5
Maximum root depth, RD_{mx} (cm)	90	90
Plant radiation-use efficiency, BE [(kg*ha^{-1})/(MJ*m^{-2})]	40	40
Harvest index, HI	0.5	0.5
Total potential heat units required for crop maturation, PHU (^oC)	2000	2100
A parameter expressing the sensitivity of harvest index to drought, WYSF	0.05	0.05

Table S3 Values of the hydrological parameters for AWPM-SG

Depths (cm)	Layer and soil type	*ms* (cm^3/cm^3)	*md* (cm^3/cm^3)	ks (cm/d)	C	dp	D0	b
		Initial values						
0-90	Loam	0.395	0.07	15	8		0.88	35.4
90-300	Sandy loam	0.43	0.07	14		0.08		
		Calibrated values						
0-90	Loam	0.395	0.02	15	13		0.1	17.5
90-300	Sandy loam	0.41	0.02	23		0.2		

Note: ms, md are the saturated moisture, residual moisture of 0-90 cm soil, respectively; ks is the saturated hydraulic conductivity; C, b are the content of soil in zone 1 and 2; D0 is the diffusion rate at wilting point; dp is the drainable porosity.

Table S4 Irrigation scheduling carried out for the experiments of maize in Shuguang village in 2007 and 2008

Year	Irrigation events	Date (month/day)	Irrigation depth(mm)
2007	First	6/26	97.5
	Second	7/17	90
	Third	8/1	97.5
	Fourth	8/22	75
2008	First	6/27	97.5
	Second	7/11	90
	Third	7/30	97.5
	Fourth	9/1	75

Table S5 Mean relative error, Root mean square error, Regression coefficient, Nash and Sutcliffe model efficiency and Coefficient of determination of the model calibration and validation

Years	Items	Groundwater depth (m)	Root mean square error, RMSE	Nash and Sutcliffe model efficiency, NSE	Mean relative error, MRE (%)	Coefficient of determination, R^2	Regression coefficient, b
2007	soil moisture(cm)	1.5a	3.47	0.5	6.83	0.54	1.01
		1.5b	3.06	0.47	-2.17	0.48	0.95
		2a	2.89	0.68	2.77	0.69	0.99
		2b	3.18	0.55	4.17	0.58	1.01
		2.5a	4.12	0.11	-5.47	0.4	0.93
		2.5b	4.68	-0.31	-11.19	0.4	0.88
		3a	4.01	-0.14	-8.92	0.48	0.91
		3b	5.46	-0.45	-5.8	0.15	0.9
	Groundwater(cm)	1.5a	14.1	0.57	-4.11	0.71	0.95
		1.5b	12.1	0.71	-1.64	0.75	0.97
		2a	8.63	0.49	-1.15	0.54	0.99
		2b	8.21	0.61	1	0.64	1.01
		2.5a	4.73	-0.77	1.03	0.35	1.01
		2.5b	20.17	-0.65	7.03	0.44	1.06
		3a	5.02	-0.59	1.47	0.6	1.01
		3b	7.39	-1.24	2.42	0.75	1.02
	LAI(cm^2/cm^2)	1.5a	0.51	0.92	-10.14	0.98	0.89
		1.5b	0.48	0.91	11.35	0.92	1
		2a	0.67	0.87	-6.37	0.9	0.89
		2b	0.35	0.96	6.87	0.97	0.97
		2.5a	0.57	0.87	20.86	0.91	1.07
		2.5b	0.78	0.81	0.69	0.84	0.89
		3a	0.33	0.96	12.66	0.98	1.06
		3b	0.48	0.91	18.48	0.96	1.1
2008	soil moisture(cm)	1.5a	3.27	0.49	12.84	0.87	1.08
		1.5b	2.18	0.68	1.4	0.7	0.99
		2a	2.94	0.54	8.66	0.68	1.05
		2b	3.86	0.47	15.82	0.74	1.08
		2.5a	3.45	0.29	16.5	0.8	1.13
		2.5b	3.43	0.36	10.4	0.51	1.06
		3a	1.8	0.8	0.87	0.81	0.99
		3b	3	0.56	10.57	0.71	1.06
	Groundwater(cm)	1.5a	17.5	-0.53	-8.28	0.61	0.91
		1.5b	17.98	-0.29	-8.21	0.61	0.91
		2a	5.54	0.46	-0.96	0.59	0.99

	2b	5.47	0.33	-0.91	0.56	0.99
	2.5a	6.8	-5.93	2.49	0.12	1.02
	2.5b	10.5	-3.52	3.96	0.14	1.04
	3a	7.65	-6.35	2.42	0	1.02
	3b	8.48	-8.65	2.74	0.01	1.03
LAI(cm^2/cm^2)	1.5a	0.44	0.88	-3.49	0.94	1.05
	1.5b	0.49	0.86	-11.4	0.92	0.98
	2a	0.8	0.57	19.88	0.9	1.22
	2b	0.69	0.62	8.62	0.96	1.2
	2.5a	0.91	0.41	29.52	0.96	1.32
	2.5b	0.9	0.29	19.11	0.93	1.29
	3a	1.03	0.13	35.61	0.98	1.4
	3b	1.01	0.22	33.29	0.94	1.36

Table S6 Uncertainty analysis of parameters for AWPM-SG

d-factor	Parameters								
	mf	ms	md	mwp	k2s	ks	C	D0	b
Groundwater depth	1.44	0.38	0.03	0.006	0.07	0.2	0.15	0	0.002
LAI	0.006	0.002	0	0.003	0	0	0	0	0
Soil water content	0.72	0.28	0.02	0.07	0.07	0.12	0.11	0	0.001
	dp	LAI_{mx}	ad	Tb	T0	kb	be	bt	PHU
Groundwater depth	0.18	0.2	0	0.38	0.07	0.03	0.08	0.008	0.45
LAI	0	0.56	0.11	0.29	0.19	0	0.003	0.008	0.39
Soil water content	0.06	0.29	0.04	0.14	0.03	0.06	0.11	0.01	0.18

Note: In the uncertainty analysis, larger *d-factor* represents larger uncertainty of parameters. mf , ms, md, mwp are the field capacity, saturated moisture, residual moisture, wilting moisture of 0-90 cm soil, respectively; k2s is the saturated hydraulic conductivity in zone 1; ks is the saturated hydraulic conductivity in zone 2; C, b are the content of soil in zone 1 and 2; D0 is the diffusion rate at wilting point; dp is the drainable porosity; LAI_{mx} is the maximum of crop leaf area index; ad is a parameter that governs LAI decline rate for crop; Tb is the minimun temperature for plant growth, T0 is the optimal temperature for plant growth; kb dimensionless canopy extinction coefficient; be, bt are the parameters for evaporation and transpiration; PHU is total potential heat units required for crop maturation.
